# Superficial Serrated Adenoma: A Rare Case of Sigmoid Polyp With Malignant Potential

**DOI:** 10.7759/cureus.76305

**Published:** 2024-12-24

**Authors:** Yu-Cong Chen, Yung-Ning Huang, Yang-Bor Lu

**Affiliations:** 1 Department of Digestive Disease, Xiamen Chang Gung Hospital, Hua Qiao University, Xiamen, CHN

**Keywords:** endoscopic mucosal resection, kras, potential malignant transformation, rspo fusion/expression, superficial serrated adenoma

## Abstract

We present the case of a 68-year-old woman who underwent complete endoscopic resection of a superficial serrated adenoma (SuSA). Due to its rarity and limited case reports, SuSA is often misdiagnosed as a hyperplastic lesion without malignant potential, leading to missed diagnoses. A polypoid lesion was identified in the sigmoid colon during the initial endoscopic evaluation, where it was initially classified as a sessile serrated lesion (SSL). The subsequent endoscopic evaluation, using crystal violet staining, revealed a type IIIL pit pattern. Endoscopic mucosal resection (EMR) was performed, and histopathological analysis confirmed serrated glandular hyperplasia with mild atypia. Immunohistochemical staining showed cytokeratin 20 (CK20) expression predominantly in the upper layer, while Ki-67 and cellular myelocytomatosis oncogene (c-MYC) were distributed in the basal and intermediate layers. Beta-catenin positivity was observed in the cytoplasm of some nuclei, confirming the diagnosis of SuSA. This case underscores the importance of timely recognition and management of SuSA to prevent progression to more severe conditions.

## Introduction

Superficial serrated adenoma (SuSA), a recently described pathological entity in 2018 [1], was not included in the World Health Organization (WHO) Fifth Edition Classification of Serrated Colorectal Lesions (2019) due to its recent discovery [2]. SuSA predominantly occurs in the sigmoid colon and exhibits distinct clinicopathological and molecular features, combining characteristics of both adenoma and serrated lesions [3]. Its atypical presentation makes it challenging to classify accurately.

## Case presentation

**Figure 1:**
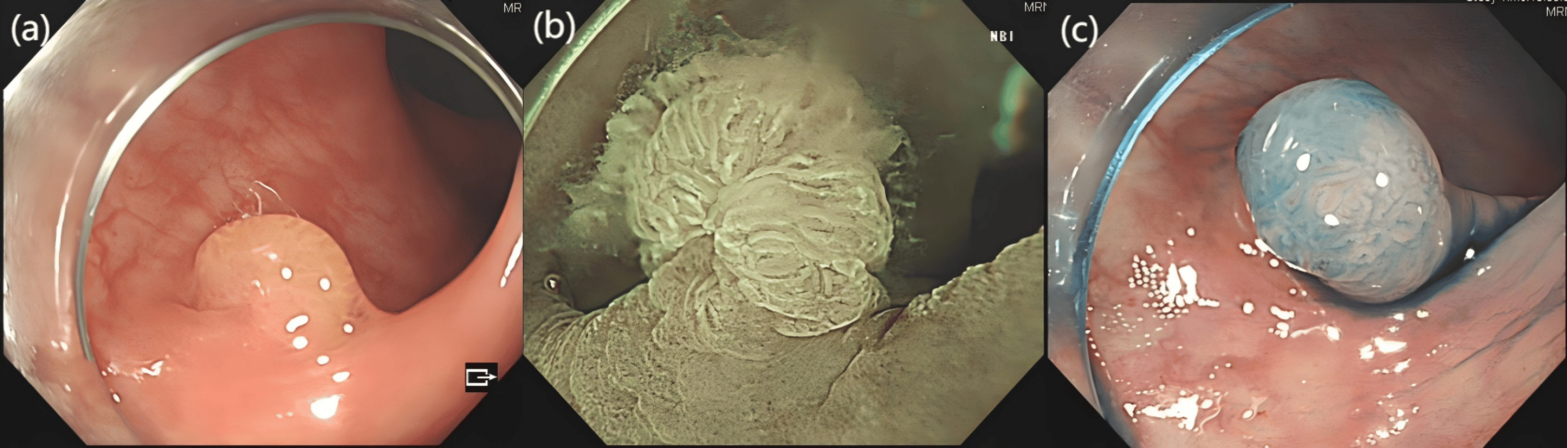
Colonoscopy Image (a) Under white light, a yellowish sessile polypoid lesion is visible. (b) With magnification in water under narrow-band imaging (NBI), mucus secretion is observed on the tumor’s surface, with glandular structures becoming prominent. (c) Following indigo carmine staining, the lesion displays a pit pattern IIIL, indicative of serrated glandular architecture.

A 68-year-old woman underwent a colonoscopy at our hospital, during which a 1.0cm x 0.8cm polyp was identified in the sigmoid colon. The initial endoscopic impression suggested a sessile serrated lesion (SSL), while preliminary pathological evaluation indicated a serrated lesion. One month later, the patient underwent endoscopic treatment. The polyps were found to have a smooth surface and regular margins. Crystal violet staining and magnifying endoscopy revealed pit pattern IIIL type, JNET 2A type, with good injection lifting (Figure 1).

The lesion was excised using high-frequency snare electrocautery without procedural complications. Post-resection histopathology confirmed the presence of serrated adenoma. Microscopic morphology and immunohistochemical results further supported the diagnosis of SuSA. Cytokeratin 20 (CK20) was strongly expressed in the upper layer, while Ki-67 was approximately 40% positive in the basal layers, and cellular myelocytomatosis oncogene (c-MYC) was positive. β-catenin positivity in the cytoplasm was also noted, aligning with the clinical judgment of SuSA.

## Discussion

**Figure 2:**
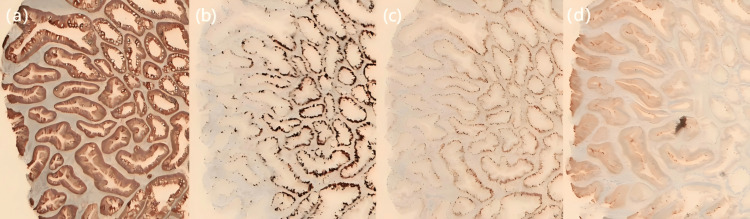
Pathological Image (a) Cytoplasmic positivity for β-catenin. (b) Weak positivity for Ki-67, localized to the basal and intermediate layers of the tissue. (c) Expression of cellular myelocytomatosis oncogene (c-MYC) is evident in the basal and intermediate layers of the tissue. (d) Positivity for cytokeratin 20 (CK20).

SuSA is a recently described entity with mixed morphological features of adenoma and serrated lesions. Histologically, it comprises predominantly adenomatous glands with serrated structures on the surface. Immunohistochemical analysis typically shows CK20 expression confined to the upper layer, while Ki-67 positivity indicated cellular proliferation in the basal and intermediate layers (Figure 2).

Endoscopically, SuSA l esions often appear white with well-defined, irregular-bordered, and a lobulated surface. Under narrow-band imaging (NBI), vascular patterns are subtle, often displaying lace-like microvessels. Pigment endoscopy frequently reveals stellate or elongated pits, indicating serrated microstructures [3,4].

Serrated polyps are broadly classified into three subtypes: hyperplastic polyps (HPs), SSLs, and traditional serrated adenomas (TSAs). SSLs are considered to be a precursor lesion of colorectal carcinoma (CRC) with microsatellite instability (MSI), whereas TSAs are associated with microsatellite stability (MSS) and molecular features of both the serrated and adenoma-carcinoma pathways. TSAs are further divided into BRAF and KRAS subtypes [5,6] . Emerging evidence suggests SuSA may present as a precursor lesion of KRAS-type TSAs, exhibiting KRAS mutations and RSPO fusion/expression [7] . ​​​​​​ These molecular traits underscore SuSA's potential for malignant transformation, emphasizing the necessity of its early detection and intervention during endoscopic procedures [1] .

## Conclusions

Here, we presented a case of complete endoscopic resection of a SuSA. Given its rarity and the limited number of case reports, SuSA is frequently misdiagnosed as a hyperplastic lesion, leading to missed opportunities for diagnoses. However, SuSA carries a risk of malignant transformation, emphasizing the need for early identification and intervention during endoscopy. This case highlights the significance of prompt recognition and management of SuSA to prevent progression to more advanced conditions.
